# Real-Time Detection of an Undercarriage Based on Receptive Field Blocks and Coordinate Attention

**DOI:** 10.3390/s23249861

**Published:** 2023-12-16

**Authors:** Ruizhen Gao, Ya’nan Ma, Ziyue Zhao, Baihua Li, Jingjun Zhang

**Affiliations:** 1 School of Mechanical Engineering and Equipment, Hebei University of Engineering, Handan 056038, China; 2 Key Laboratory of Intelligent Industrial Equipment Technology of Hebei Province, Hebei University of Engineering, Handan 056038, China; 3 Collaborative Innovation Center for Modern Equipment Manufacturing of Jinan New Area (Hebei), Handan 056038, China; 4 Department of Computer Science, Loughborough University, Loughborough LE11 3TU, UK

**Keywords:** small targets detection, YOLOv5, receptive field block, coordinate attention mechanism, loss function

## Abstract

Currently, aeroplane images captured by camera sensors are characterized by their small size and intricate backgrounds, posing a challenge for existing deep learning algorithms in effectively detecting small targets. This paper incorporates the RFBNet (a coordinate attention mechanism) and the SIOU loss function into the YOLOv5 algorithm to address this issue. The result is developing the model for aeroplane and undercarriage detection. The primary goal is to synergize camera sensors with deep learning algorithms, improving image capture precision. YOLOv5-RSC enhances three aspects: firstly, it introduces the receptive field block based on the backbone network, increasing the size of the receptive field of the feature map, enhancing the connection between shallow and deep feature maps, and further improving the model’s utilization of feature information. Secondly, the coordinate attention mechanism is added to the feature fusion network to assist the model in more accurately locating the targets of interest, considering attention in the channel and spatial dimensions. This enhances the model’s attention to key information and improves detection precision. Finally, the SIoU bounding box loss function is adopted to address the issue of IoU’s insensitivity to scale and increase the speed of model bounding box convergence. Subsequently, the Basler camera experimental platform was constructed for experimental verification. The results demonstrate that the AP values of the YOLOv5-RSC detection model for aeroplane and undercarriage are 92.4% and 80.5%, respectively. The mAP value is 86.4%, which is 2.0%, 5.4%, and 3.7% higher than the original YOLOv5 algorithm, respectively, with a detection speed reaching 89.2 FPS. These findings indicate that the model exhibits high detection precision and speed, providing a valuable reference for aeroplane undercarriage detection.

## 1. Introduction

With the gradual development of machine vision, deep learning-based target detection tasks are gaining attention, supporting various essential field applications such as face detection, industrial anomaly detection, and gesture recognition. An image sensor, widely used in digital cameras and other electro-optical devices, converts optical image information into electrical signals. In image detection tasks, reliance on the camera sensor captures image data for the corresponding task. Different types of cameras can be chosen to obtain images with varying resolutions, color information, and other conditions. This combination not only aids the sensor system in understanding the contents of its monitoring environment more accurately, but also allows for the independent selection of different image data for corresponding processing. Furthermore, a deep learning image detection model, capable of identifying unusual activities, can trigger alarms or take appropriate measures through the sensor system. Introducing these models into the sensor system enables faster decision-making, assists in more informed actions, and reduces energy consumption, thereby extending the life of the sensor equipment. The rapid evolution of artificial intelligence is driving the integration of machine vision and image sensor chips, giving rise to an “intelligent image sensor” that will shape the pulse and direction of technological development.

Aeroplanes are crucial in daily travel, cargo transportation, military surveys, and operations. Ensuring aeroplane survivability for safe air travel is a recurring consideration for engineers. Currently, many airplane crashes occur during takeoff and landing [1]. Whether or not the undercarriage is properly deployed or folded, and whether or not the undercarriage is detached from the fuselage during liftoff and landing, can affect the pilot’s ability to maneuver the aeroplane properly. As a result, real-time detection of the undercarriage’s status is a critical consideration. However, the small size of the undercarriage results in low detection precision and poses a challenge to existing detection algorithms. Effectively addressing the issue of real-time undercarriage detection for small targets has become an urgent concern.

With the maturation of computer vision, target detection algorithms are categorized into two-stage algorithms and one-stage algorithms based on the presence or absence of a separate candidate frame screening stage. Two-stage algorithms, including the region-based convolutional network R-CNN [2], fast regional convolutional network Fast R-CNN [3], faster regional convolutional network Faster R-CNN [4], and mask regional convolutional network Mask R-CNN [5]. The two-stage algorithm improves the localization precision of the targets. However, their multiple detection and classification operations require longer computation times and lower detection speeds. On the other hand, one-stage algorithms, such as RetinaNet [6], EfficientDet [7], Single-shot Multi-frame Detection (SSD) [8], and You Only Look Once (YOLO) [9,10,11,12], directly output category probability, resulting in high detection speed.

Deep learning-based target detection algorithms contribute to various applications, including remote sensing image detection [13,14,15], defect detection [16,17], targets tracking [18,19], and face recognition [20,21]. Among them, the YOLO series of algorithms is the most widely used. In the medical field, Doniyorjon et al. [22] improved the precision of wireless endoscope image detection by adding ResNeT to the YOLOv4 backbone network. Liu [23] used a multiscale fusion network, bidirectional feature pyramid, and YOLOv5-based EIOU loss function to improve the precision of pulmonary nodule detection in CT images. In fruit detection, Ji et al. [24] introduced a lightweight network and PAN feature pyramid in the YOLOv4 algorithm, reducing the model size and improving the speed of detecting fruits. Xu et al. [25] enhanced the YOLOv5 algorithm by introducing the DIOU loss function and SE attention mechanism to improve apple detection precision. For remote sensing monitoring, Li et al. [26] improved the detection precision of small-resolution images by incorporating an attention mechanism into YOLOv5, a bidirectional feature pyramid, and a small-targets detection layer. In contrast, Su et al. [27] lightened the YOLOv5 module and optimized the loss function to enhance the network’s detection performance for remote sensing images.

You Only Look Once (YOLO) is a one-stage object detection algorithm that prioritizes detection speed, leading to low recall. YOLOv1 employs regression to perform direct classification and prediction tasks using first-order networks. YOLOv2 improves on YOLOv1 by abandoning GoogleNet, adopting Darknet-19 with fewer convolutions as a feature extraction network to enhance detection performance, and introducing a priori frame to improve the recall rate. YOLOv3 further updates the feature extraction network to Darknet-53 with a multiscale framework, incorporating a residual structure to increase depth and output three feature maps of different scales for object detection, thereby improving the detection precision of small targets. YOLOv4 designs a feature extraction network based on the Darknet53 cross-stage section (CSP) structure to form the backbone network, reducing computation and enhancing gradient performance. It introduces a spatial pyramid pooling module to the last layer of the backbone network to address possible information distortion during network propagation. Ciou-loss and Mish activation functions are introduced to enhance detection precision further. In 2020, the Ultralytics team proposed YOLOv5, achieving a 55.4% mAP index on the MS COCO dataset. This demonstrated stronger detection performance than YOLOv4 and increased suitability for real-time detection of aeroplanes and undercarriages. Therefore, this paper proposes the YOLOV5-RSC net model based on YOLOv5s to improve the detection precision targeting airplanes and landing gears. The main contributions of this paper are shown as follows:

1. Adding a Receptive Field Block to the backbone network to improve the feature map receptive field and fully extract multiscale features.
2. Adding a coordinate attention mechanism to the feature fusion network to enhance the model’s focus on key information and improve the model’s detection precision.
3. Introducing the SIOU loss function to accelerate model convergence and further improve model detection performance.
4. Construct a dataset to validate the reliability and scientific validity of the improved model and design multiple sets of ablation experiments to verify the validity of each module based on the improvement points.

## 2. Related Work

### 2.1. Definition of Small Targets and Challenges

With the rapid development of target detection algorithms, their performance and speed have significantly improved. However, due to the small proportion of objects detected in some images, the lack of sufficient appearance information makes it challenging for the network to obtain enough feature information when extracting features, ultimately leading to unsatisfactory detection performance. Detecting small targets remains a challenging problem in the field of target detection. Currently, two ways of defining small targets are based on absolute and relative scales. Based on the absolute scale, Torralba’s team [28] demonstrated through research that, for most images, the pixel size of the image that people can effectively identify is 32 × 32. It is difficult for people to identify pixels below this size accurately; therefore, if the pixel size of the image is smaller than 32 × 32, it is considered a small target. Chen et al. [29] started from the relative scale, arguing that if the detection targets occupy between 0.08% and 0.58% of the whole image, it is a small target. Considering that defining small targets based on the relative scale is prone to the interference of data preprocessing, which has a certain impact on evaluating the detection performance of the model, this paper starts from the absolute scale and defines image pixels smaller than 32 × 32 as small targets.

Currently, the problems and challenges faced by target detection algorithms in small targets detection mainly include the following three points: mainstream detection algorithms are usually for targets above the mesoscale, with less optimization for small targets, resulting in poor detection performance; the low percentage of small targets pixels makes it difficult for the network to extract feature information from small target fully; and the existing dataset has very few small targets, making it difficult to uniformly distribute them in the dataset, thus affecting the network detection performance.

### 2.2. Small Targets Detection Methods

For small targets detection, one can start from the input data and use data enhancement operations, which can increase the number of small and medium targets detected, mainly including scaling [30], random cropping [31], mosaic enhancement [12] and other methods. With the help of instance segmentation, Chen et al. [32] proposed a segmentation network to replicate the target region in context to solve the problem of scale mismatch in the replication process to improve the data enhancement effect.

Multiscale learning can enhance the network’s ability to link between shallow and deep information. The FPN feature pyramid network proposed by T. Y. Lin’s team [33] takes into account that different feature maps correspond to different perceptual fields and express varying information. Fusing feature maps of different levels can enhance the network’s ability to mine features and improve the model’s performance detecting small targets. In 2014, Goodfellow et al. [34] introduced the generative adversarial network (GAN), consisting of two mutually adversarial sub-networks: the generator and discriminator networks. In small target detection, GAN reduces the feature differences between small and other targets by converting low-resolution targets into corresponding high-resolution features. This process helps express the feature information of small targets more clearly, thereby improving the effectiveness of small target detection.

Additionally, due to the design of the anchor frame in the target detection task, the samples of small and large targets are extremely imbalanced. This imbalance causes the network to pay too much attention to large targets, neglecting small target detection. Therefore, the anchor frame-free mechanism has recently become a research hotspot. Yang et al. [35] proposed a detection method called Representative Points (RepPoints), allowing the network to learn spatial and semantic information automatically, improving the precision of small target detection. Kong et al. [36] introduced a method to directly predict the probability of target existence and the bounding box coordinates. The network gains robustness and generalization ability for small targets by removing the anchor box’s restriction.

### 2.3. Problems with Undercarriage Inspection Systems

In aeroplane take-off and landing, its speed dynamically changes, impacting the state of the undercarriage. If undercarriage information is not timely feedback, the system’s impact on detection precision will be significant. Hence, the detection system must meet real-time requirements to deliver prompt information. Different aeroplane types have distinct rollers, and the structure and size of the aeroplane rollers will affect undercarriage detection precision, potentially leading to system misjudgment. In addition, the distance between the camera sensor and the undercarriage is a critical factor influencing detection precision. As the shooting distance increases, the resolution of the targets of interest in the image decreases. The lighting changes in the environment become more drastic, reducing the visibility of the undercarriage in the image. This reduction in visibility prevents the detection system from accurately capturing the important details of the undercarriage, thus affecting detection precision. Therefore, a real-time detection system for small targets is necessary to meet the real-time monitoring requirements of the undercarriage status.

### 2.4. Research Object

Because no aeroplane type comes into contact with people more often in their daily lives than passenger aeroplane, this paper mainly focuses on the acquisition of the dataset and detection of the category of the passenger aeroplane. In this category, the undercarriage mostly follows a three-point structure, with a length of about 14 m, a width of about 4 m, and roller diameters of about 400 mm.

## 3. Methodology

### 3.1. YOLOV5-RSC Model Structure

**Figure 1:**
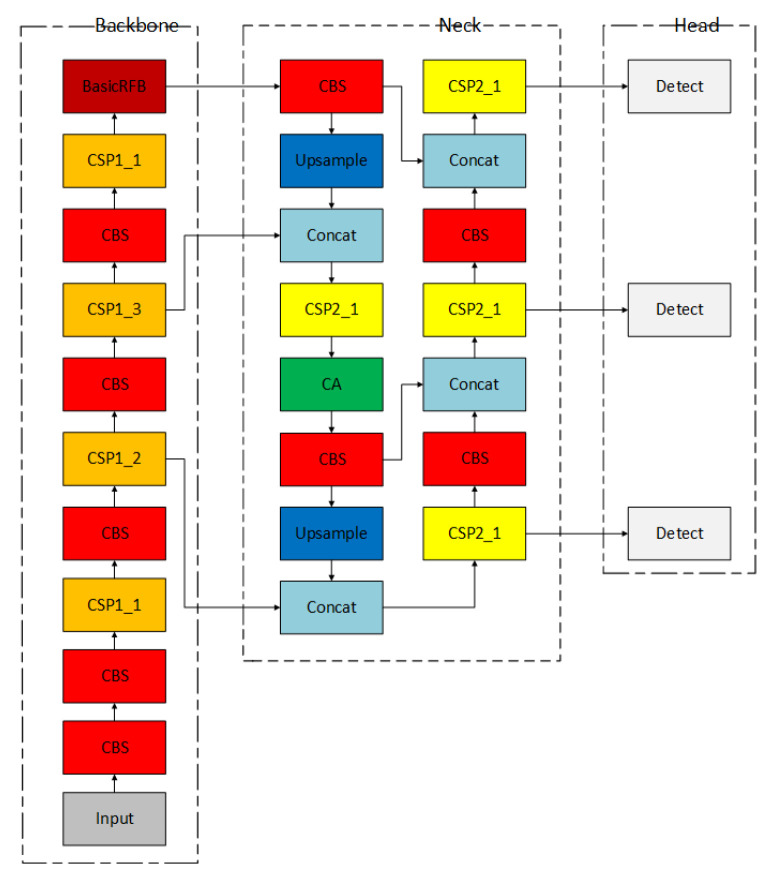
YOLOv5-RSC structure.

The YOLOv5-RSC model consists of the backbone network, feature extraction network, and detection head. The backbone network includes a convolutional layer, CSP1-X residual network, and Receptive Field Block, which are responsible for extracting input data features and increasing network depth. The feature extraction network comprises a convolutional layer, CSP2-X network, concat module, and Coordinate Attention Mechanism, aiming to fuse features from the trunk network and enhance network detection performance. The detection head includes three convolutional modules of different sizes designed to output detection results. Previous efforts in optimizing the YOLO algorithm have focused on lightweight design, introducing attention mechanisms, and modifying loss functions to enhance detection precision. However, these optimizations have not significantly improved the detection of small targets. To address this, this article proposes the YOLOv5-RSC network model specifically to enhance the detection performance of small targets, particularly in detecting aeroplane undercarriages. The proposed model introduces a Receptive Field Block module, utilizing different rates to represent convolutional layer parameters. This module concatenates outputs of convolutional layers with different sizes and rates to fuse features and strengthen contextual connections. The coordinate attention mechanism is also incorporated to improve the network’s focus on key information and suppress irrelevant details, thereby enhancing detection precision. Ablation experiments are conducted to determine the optimal insertion position of the coordinate attention mechanism. Finally, the original bounding box loss function CIOU in the YOLOv5-RSC network is replaced with SIOU to expedite model convergence and improve precision. The framework of the improved YOLOv5-RSC model is illustrated in Figure 1.

### 3.2. Receptive Field Block

**Figure 2:**
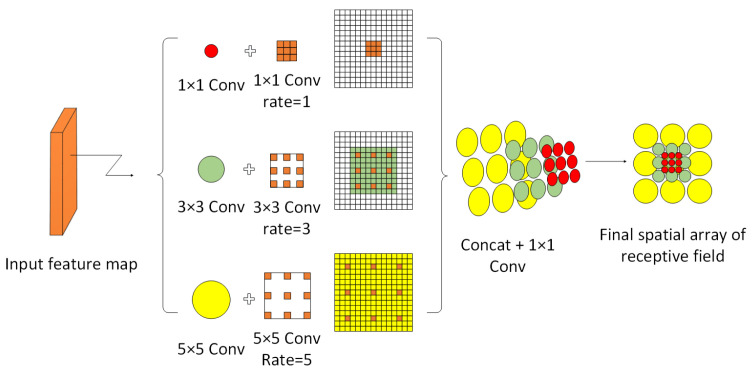
Receive Field Block schematic diagram.

In order to allow the network to improve the receptive field of each layer of the feature map while maintaining the same number of parameters and generating a larger resolution feature map, and to improve the ability of the network to mine fine features of small targets while also avoiding the confusion of contextual information, Liu et al. [37] proposed the Receptive Field Block (RFB). The starting point of this paper is to simulate the receptive field of human vision in order to build a spatial array, and Figure 2 shows the schematic diagram of RFB.

RFB references the Inception structure; the first layer of each branch is composed of 1 × 1, 3 × 3, 5 × 5 convolutional kernels, and the convolutional layers of different sizes of convolutional kernels are combined to splice into a multi-branch structure to obtain different receptive fields and make the network have a multiscale perception of the input data, which makes it easier to grasp information at different scales; in addition, the Deeplab algorithm’s Dilated Convolution is introduced to expand the network’s acceptance domain while maintaining image resolution, enabling accurate targets positioning while acquiring multiscale features. After the Dilated convolution processes the data, all branches are Concated together in the channel dimension and subjected to 1 × 1 convolution. Then, the data are merged with that of another branch, and finally, output by the RELU activation layer, and the final output of RFB is concatenated with feature maps of different sizes to achieve the purpose of feature fusion.

Based on the principle of RFBNet, we introduced it into the YOLOv5-RSC model proposed in this article, changing the original spatial pyramid pooling module SPPF to BasicRFB.

### 3.3. SIOU Loss Function

The loss function measures the difference between the actual variable values and the predicted values. The SIOU loss function proposed by Rezatofighi et al. [38] takes into account the vector angle relationship between the prediction frame and the true frame, and adding an angle penalty term to the loss function allows the prediction frame to deviate to the nearest axis at a faster rate, thus reducing the inference time and the number of degrees of freedom. SIOU consists of angle loss, distance loss, shape loss, and IoU loss in four basic loss functions.

#### 3.3.1. Angle Loss

**Figure 3:**
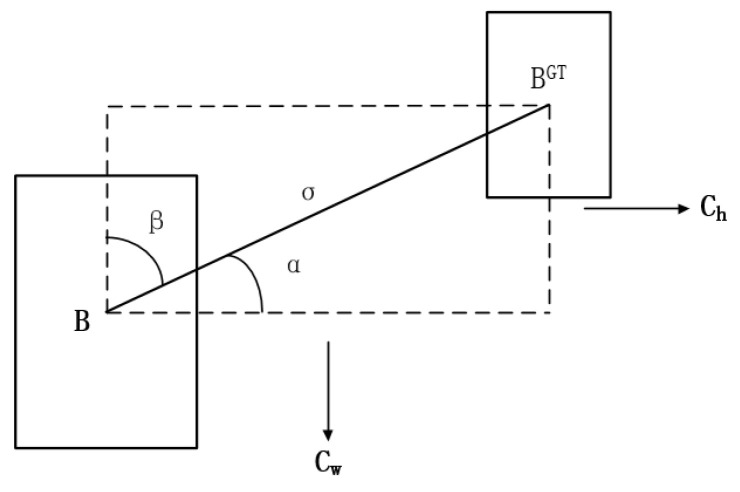
Angle loss schematic diagram.

The angle loss function reduces the number of variables in the distance-related “wonder” by first pulling the prediction to the closest axis (X/Y axis) and then extrapolating along this axis to approximate the true frame, as shown in Figure 3.

When $\alpha \leq \frac{\pi }{4}$, try to minimize α, otherwise minimize $\beta =\frac{\pi }{4}-\alpha$; see Equations (1)–(3) for the calculation.

(1) $$\wedge =1-2\sin^{2}\left(\arcsin\left(\frac{c_{h}}{\sigma }\right)-\frac{\pi }{4}\right)$$

(2) $$\sigma =\sqrt{{\left(b_{cx}^{gt}-b_{cx}\right)}^{2}+{\left(b_{cy}^{gt}-b_{cx}\right)}^{2}}$$

(3) $$ch=max\left(b_{cy}^{gt},b_{cy}\right)-min\left(b_{cy}^{gt},b_{cy}\right)$$

where σ is the distance between the center point of the real box and the center point of the predicted box, and ch is the height difference between the center point of the real box and the center point of the predicted box. $b_{cx}$ and $b_{cy}$ is the center point coordinate of the prediction box. $b_{cx}^{gt}$ and $b_{cy}^{gt}$ is the center point coordinate of the real box.

#### 3.3.2. Distance Loss

Redefining distance loss on the basis of angular loss.

(4) $$△=\sum_{t=x,y}^{}\left(1-e^{-\gamma \rho t}\right)=2-e^{-\gamma \rho_{x}}-e^{-\gamma \rho_{y}}$$

(5) $$\rho_{x}={\left(\frac{b_{cx}^{gt}-b_{cx}}{c_{w}}\right)}^{2},\rho_{y}={\left(\frac{b_{cy}^{gt}-b_{cy}}{c_{h}}\right)}^{2},\gamma =2-\wedge$$

**Figure 4:**
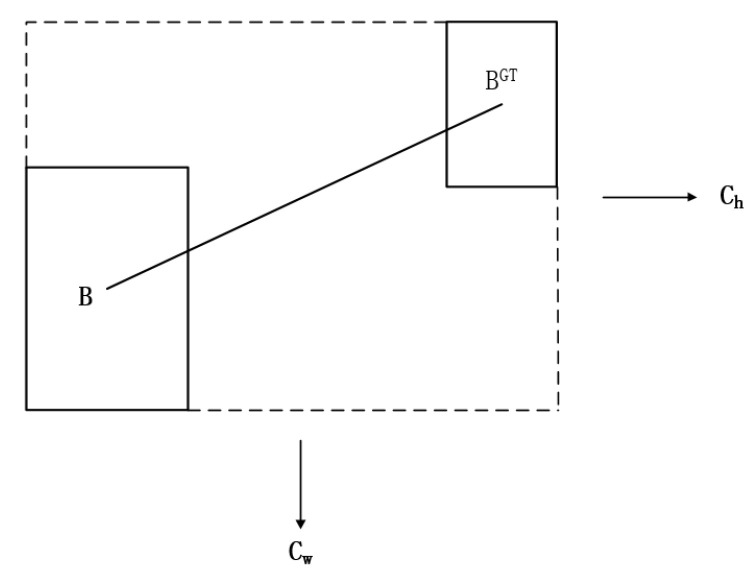
Distance loss schematic diagram.

$c_{w}$ and $c_{h}$ represent the width and height of the minimum outer rectangle of the real box and the predicted box, respectively. When α→0, the smaller the weight of Δ, and when $\alpha \to \frac{\pi }{4}$, the larger the weight of Δ, the greater the angle will make the problem more difficult, so assign γ a time-limited distance value and the scheme to calculate the distance between the real frame and its prediction is shown in Figure 4.

#### 3.3.3. Shape Loss

(6) $$\Omega =\sum_{t=w,h}^{}{\left(1-e^{-\varpi t}\right)}^{\theta }={\left(1-e^{-\varpi_{w}}\right)}^{\theta }{\left(1-e^{-\varpi_{h}}\right)}^{\theta }$$

(7) $$\varpi_{w}=\frac{\left|w-w^{gt}\right|}{\max\left(w,w^{gt}\right)},\varpi_{h}=\frac{\left|h-w^{gt}\right|}{\min\left(h,h^{gt}\right)}$$

where *w*, *h*, $w^{gt}$, and $h^{gt}$ are the predicted box’s width and height, respectively. θ controls the level of attention to shape loss. In order to avoid paying too much attention to shape loss and impacting the prediction frame’s action, the authors use the genetic algorithm to give the range of θ parameters as [2, 6].

#### 3.3.4. IoU Loss

**Figure 5:**
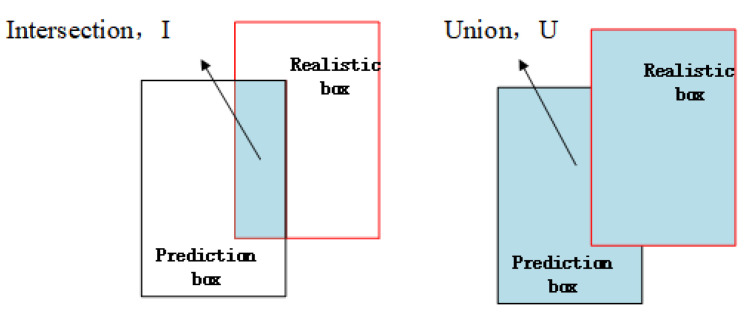
IOU calculation schematic diagram.

IoU is the cross-merge ratio between the real frame and the predicted frame. The principle is shown in Figure 5, and the calculation is shown in the equation:

(8) $$IoU=\frac{\left|I\right|}{\left|U\right|}$$

*I* and *U* are the intersection and union regions of the real and predicted boxes, respectively. Combining the four loss functions yields the final loss function $L_{box}^{siou}$; see Equation (9).

(9) $$L_{box}^{siou}=1-IoU+\frac{\Omega +△}{2}$$

#### 3.3.5. Introducing the SIOU Loss Boundary Box Loss Function

The YOLOv5 loss consists of the confidence loss Lobj classification loss Lcls and the bounding box loss Lbox, and the weighted sum of the three constitutes the final loss; see Equation (10).

(10) $$L_{all}=\sigma_{1}L_{obj}+\sigma_{2}L_{cls}+\sigma_{3}L_{box}$$

where $\sigma_{1}$$\sigma_{2}$$\sigma_{3}$ is the weight coefficient of the corresponding loss. Where the confidence loss is obtained from a binary cross-entropy calculation of the confidence score *q*${}_{0}$ of the prediction frame and the iou values qiou of the prediction frame and the true frame; see Equation (11).

(11) $$L_{obj}\left(q_{0},q_{iou}\right)=BCE_{obj}^{sig}\left(q_{0},q_{iou};w_{obj}\right)$$

BCE is the cross entropy function. The classification loss and confidence loss are similar. See Equation (12).

(12) $$L_{cls}\left(a_{p},a_{gt}\right)=BCE_{cls}^{sig}\left(a_{p},a_{gt};w_{cls}\right)$$

YOLOv5’s bounding box loss is CIOU, which introduces the concept of bounding box aspect ratio based on DIOU, considering the loss of length and width, making the predicted box more in line with the true box, as calculated below.

(13) $$L_{box}^{ciou}=1-CIOU=1-IoU+\frac{\rho^{2}\left(b,b^{gt}\right)}{c^{2}}+\alpha v$$

(14) $$v=\frac{\pi }{4}{\left(\arctan\frac{w^{gt}}{h^{gt}}-\arctan\frac{w}{h}\right)}^{2}$$

(15) $$\alpha =\frac{v}{\left(1-IoU\right)+v}$$

where $\frac{\rho^{2}\left(b,b^{gt}\right)}{c^{2}}$ is a regularization term introduced from DIOU to prevent overfitting of the model. C is the Euclidean distance between the two diagonal vertices of the smallest rectangular frame, v is used to measure the consistency of aspect ratio, and α is the weight function. This paper replaces the CIOU loss with SIOU loss to accelerate the network convergence and improve the network detection performance for stronger generalization. Equation (16) reveals the network’s final loss function.

(16) $$L_{all}^{{}^{\prime }}=\sigma_{1}L_{obj}+\sigma_{2}L_{cls}+\sigma_{siou}L_{box}^{siou}$$

### 3.4. Coordinate Attention Mechanism

#### 3.4.1. Principle of Coordinate Attention Mechanism

**Figure 6:**
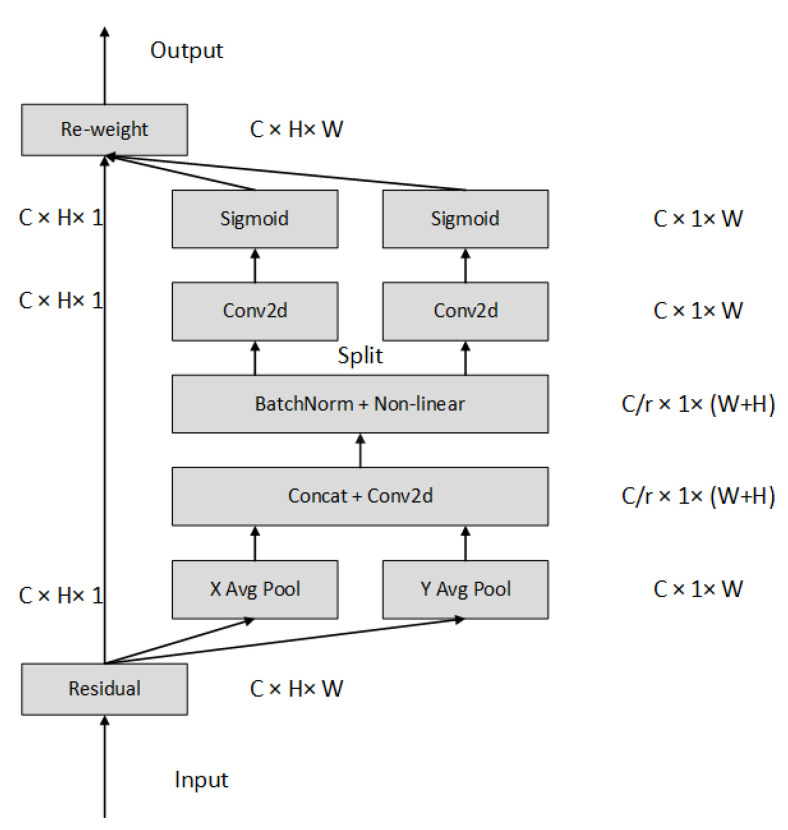
Coordinate attention structure diagram.

The attention mechanism has been developed from human vision research and is now widely used in computer vision. Its main role is to help the network focus on critical information with high weight and suppress irrelevant information with low weight, which solves the information overload problem that may occur in the model and improves the efficiency of the network processing task. In order to solve the problem whereby the Convolutional Block Attention Module (CBAM) [39] is weak in extracting information from long distances, the Coordinate Attention Mechanism (CA) proposed by Hou et al. [40] The coordinate attention mechanism can capture the direction and coordinate information across channels, which helps the network to locate the object of interest more accurately and improves the network detection precision. The overall structure of CA is shown in Figure 6.

CA encodes channel relationships and long-term dependencies through the location information of data, which can be divided into two steps: Coordinate information embedding and Coordinate Attention generation. In Coordinate information embedding, global pooling is used to globally encode spatial information, considering that global pooling will compress global spatial information into channel descriptors and location information cannot be preserved in order for the attention module to capture remote spatial interactions with precise location information, CA borrows Squeeze from SE Block; see Equation (17). Additionally, decompose global pooling to derive a pair of direction-aware feature maps, thereby enhancing the network’s precision in pinpointing the targets of interest.

(17) $$Z_{c}=\frac{1}{HW}\sum_{i=1}^{H}\sum_{j=1}^{W}x_{c}\left(i,j\right)$$

For input X, first encode each channel along the horizontal and vertical coordinates using a pooling kernel of size (*H*, 1) or (1, *W*) to obtain the feature maps in the height and width directions, respectively. See Equations (18) and (19).

(18) $$Z_{c}^{h}\left(h\right)=\frac{1}{W}\sum_{0\leq i\leq W}^{}x_{c}\left(h,j\right)$$

(19) $$Z_{c}^{w}\left(w\right)=\frac{1}{H}\sum_{0\leq j\leq H}^{}x_{c}\left(j,w\right)$$

where $Z_{c}$ indicates the output associated with the cth channel. For the Coordinate Attention generation operation, the two feature maps from the previous step are first concatenated according to Equation (20). Their size is reduced to *C*/*r* using a 1 × 1 size convolution kernel. After batch normalization, the feature map F1 is fed into the activation function to obtain a 1×(W+H)×C/r size feature map.

(20) $$f=\delta \left(F_{1}\left(\left[z^{h},z^{w}\right]\right)\right)$$

where $f\in R^{C/r\times \left(H+W\right)}$ denotes the feature mapping, r represents the shrinkage rate, δ is the activation function, and […, …] denotes the concatenation along the spatial dimension. *f* is then decomposed into two tensors $f^{h}$ and $f^{w}$ by spatial orientation. Two 1 × 1 convolutions $F_{H}$ and $F_{W}$ are used to act on $f^{h}$ and $f^{w}$, respectively, to obtain a tensor with the same number of channels as the input X, which is transported to Sigmoid activation function to obtain the attention weights of height and width; see Equations (21) and (22).

(21) $$g^{h}=\sigma \left(F_{h}\left(f^{h}\right)\right)$$

(22) $$g^{w}=\sigma \left(F_{w}\left(f^{w}\right)\right)$$

Finally, the feature map *Y* with height and width attention weights is obtained by multiplying and weighting Equation (23).

(23) $$y_{c}\left(i,j\right)=x_{c}\left(i,j\right)g_{c}^{h}\left(i\right)g_{c}^{w}\left(j\right)$$

#### 3.4.2. Adding Coordinate Attention

**Figure 7:**
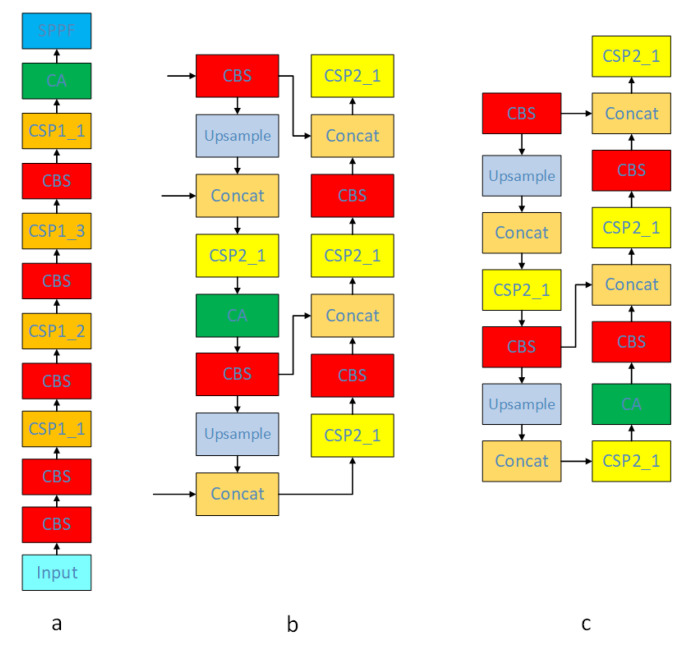
Map of the insertion position of the coordinates attention. (**a**): Insert at the front of the network; (**b**): Insert in the middle of the network; (**c**): Insert at the end of the network.

The coordinate attention is introduced into the feature fusion module in the YOLOv5 algorithm to improve the network’s attention to the object of interest and reduce the weight of irrelevant factors in the image to improve the model detection performance, combined with the study of coordinate attention insertion positions by Guo et al. [41] In order to mitigate the overfitting phenomenon arising from insufficient information generalization by the model’s channel weights and spatial weights, the insertion positions have been stratified into three distinct groups for comprehensive examination in this study. Figure 7 illustrates the insertion positions, while Table 1 presents the experimental results.

Based on the experimental results, we opted for group b as the insertion location for Coordinate Attention, given its substantial enhancement in the model’s detection precision.

## 4. Experiments and Results

### 4.1. Dataset

The image data used in this paper is sourced from various aeroplane categories within the COCO128 and VOC datasets, along with images captured at airports, amounting to 3000 images. Through data augmentation, the dataset was expanded to include 10,000 images. We utilized the image annotation tool LabelImg to annotate the aeroplane and undercarriage data. The annotated data were divided into training, validation, and test sets at 6:2:2.

### 4.2. Experimental Environment and Parameter Configuration

**Figure 8:**
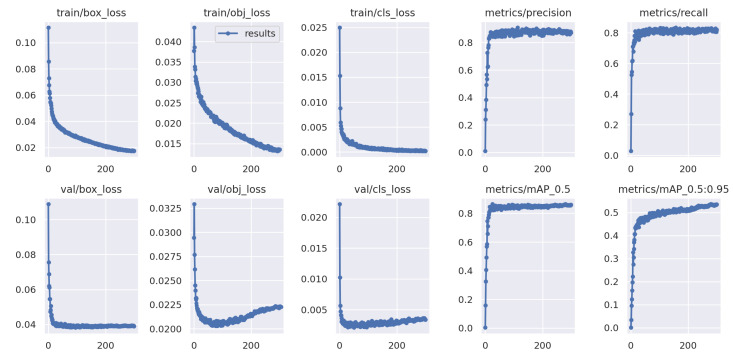
Training results’ performance metrics.

This paper implemented the YOLOv5-RSC model using the PyTorch 1.12.0 framework, leveraging CUDA version 10.2 for accelerated training. The programming language utilized was Python-3.7, and the experiments were executed on Ubuntu 18.04, employing a CPU model of Core i7 6850 K and a GPU configuration consisting of two GeForce GTX 1080Ti with 11 GB of video memory. In the experiment, YOLOv5s.pt was selected as the pre-training weight, hyperparameter evolution was enabled, and the input image size was set to 640 × 640. The initial learning rate, momentum, epoch, and batch size were configured as 0.01, 0.937, 300, and 32, respectively. After conducting 300 training rounds, we acquired the training results for the YOLOv5-RSC model, with the corresponding metrics depicted in Figure 8.

### 4.3. Evaluation Indicators

The evaluation metrics in this paper include four categories: Precision (Pr), Recall (Re), Average Precision (AP), and Mean Average Precision (mAP). Before introducing the metrics, the following concepts are given according to the confusion matrix (see Table 2): true positive (TP) is the number of correctly assigned positive samples by the classifier, true negative (TN) is the number of correctly assigned negative samples, false positive (FP) is the number of misclassified positive samples, and false negative (FN) is the number of misclassified negative samples.

Precision represents the proportion of samples with positive outcomes that are true positive cases, and recall represents the proportion of samples with true positive outcomes that are predicted positive cases, both defined according to the following formula.

(24) $$P_{r}=\frac{TP}{FP+TP}$$

(25) $$R_{e}=\frac{TP}{FN+TP}$$

Considering that precision and recall have an interactive relationship and cannot be directly involved in the evaluation, the concepts of Average Precision (AP) and Mean Average Precision (mAP) are introduced, and the higher the two indicators are, the better the detection is; see Equations (26) and (27).

(26) $$AP=\int_{0}^{1}Pr\left(Re\right)d\left(Re\right)$$

(27) $$MAP=\frac{1}{N}\sum_{i=1}^{N}AP_{i}$$

where *N* is the number of detection categories. Frames Per Second, (FPS) indicates the number of image frames that a system can process and display in one second and is a common measure of real-time performance in areas such as graphics rendering and video processing. A higher FPS means the system can process images faster, thus providing a smoother visual experience.

### 4.4. Comparison Experiments

To validate the reliability of the model proposed in this paper, the YOLOv5-RSC model was compared with several target detection algorithms on the same dataset. These algorithms include SSD, Faster R-CNN, RetinaNet, YOLOv4, and YOLOv5, using evaluation metrics such as AP values for aeroplane and undercarriage, mAP values, and FPS. The experimental results are presented in Table 3.

Based on the data results, it is evident that the YOLO series algorithms outperform other algorithms in terms of detection precision and computational speed. The mAP of the YOLOv5s model is 13.2% higher than that of the YOLOv4 algorithm, and the detection speed is increased by 30.0%. With the addition of the coordinate attention mechanism, perceptual wild block, and SIOU loss function, aeroplane and undercarriage precision are enhanced. Particularly noteworthy is the 5.4% improvement in the AP value of the undercarriage compared to the original YOLOv5 algorithm, indicating that the YOLOv5-RSC model exhibits improved performance in small target detection. Considering the Persistence of Vision effect experienced by the human eye, films are typically presented at 24 frames per second, a rate considered adequate for human visual perception. However, the YOLOv5-RSC model proposed in this article achieves FPS values close to 90 during detection, and is unaffected by computational delays in daily use. Therefore, it can meet the real-time requirements of the detection system. We compared the data of the improved model with the undercarriage detection model proposed by Gao et al. [42]. It is evident from the experimental results that our proposed model surpasses Gao’s model in terms of AP, mAP, and FPS metrics, demonstrating the effectiveness of YOLOv5-RSC. Table 4 presents the comparative data.

### 4.5. Ablation Experiments

The model in this paper performs ablation experiments on the dataset to verify the effectiveness of each module and module combination on the model, and the specific experimental data are shown in Table 5.

The table shows that the original YOLOv5 model achieved a detection Precision of 86.9%, recall of 80.0%, mAP of 82.7%, and FPS of 100 frames/s. After incorporating BasicRFB to enhance the global sensory field size of the network, the model’s Precision improved by 0.8%, recall by 0.9%, mAP by 2.3%, and detection speed decreased by 6.9 frames/s. The addition of the SIOU loss function and coordinate attention mechanism individually enhanced the model’s Precision by 0.6% and 0.8%, recall by 1.1% and 0.5%, and mAP by 2% and 2.1%, indicating that each improvement point contributed to the enhancement of the model’s detection performance. Subsequent ablation experiments reveal that the combination of the improved model enhances all model metrics, with a slight decrease in FPS. Ultimately, the Precision of the YOLOv5-RSC model proposed in this article was 89.5%, the recall rate was 82.1%, and the mAP was 86.4%. Compared with the original YOLOv5s model, each indicator improved by 2.6%, 2.1%, and 3.7%, respectively. Although the FPS is reduced by 10.8 frames/s, it can still meet the real-time targeting requirements for aeroplane and undercarriage. The detection performance of YOLOv5-RSC surpasses the original YOLOv5 algorithm when considering all metrics together.

### 4.6. Analysis of Test Results

**Figure 9:**
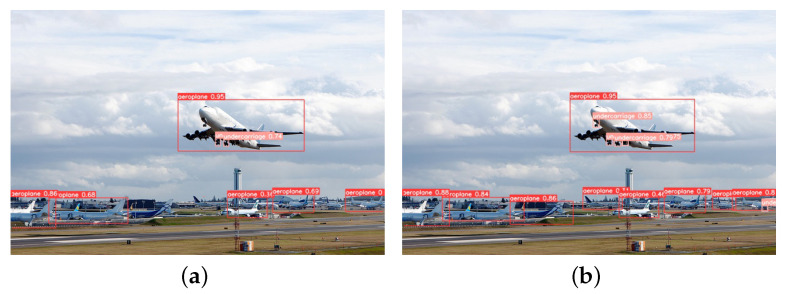
Long-range targets detection. (**a**) YOLOv5 detection results; (**b**) YOLOv5-RSC detection results.

**Figure 10:**
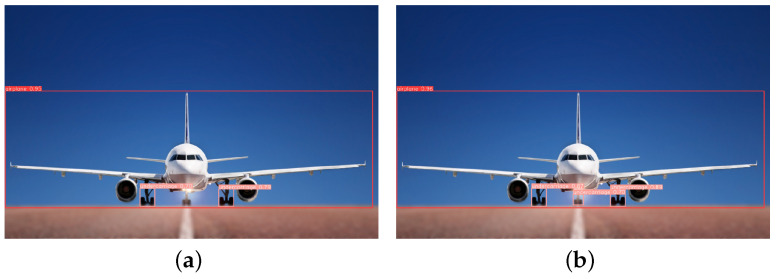
Aeroplane detection on landing. (**a**) YOLOv5 detection results; (**b**) YOLOv5-RSC detection results.

**Figure 11:**
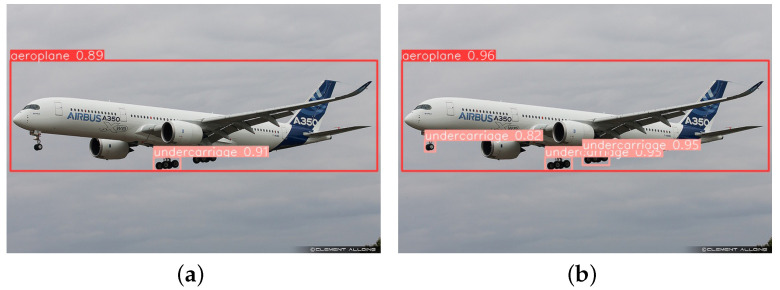
Aeroplane detection at take-off. (**a**) YOLOv5 detection results; (**b**) YOLOv5-RSC detection results.

**Figure 12:**
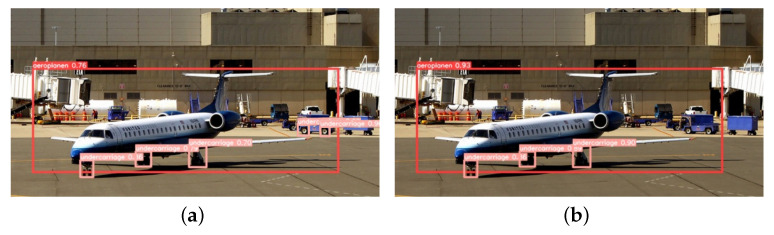
Detection inside the airfield. (**a**) YOLOv5 detection results; (**b**) YOLOv5-RSC detection results.

This paper tests several images with diverse backgrounds to validate the model’s effectiveness. Figure 9 illustrates the detection of long-distance targets, from which it can be seen that YOLOv5-RSC detects the undercarriage that is not detected by the original model, and the confidence level of the targets is higher than that of the original model, which improves the performance of long-distance undercarriage detection. Figure 10 shows the comparative detection of the aeroplane during landing. Due to the influence of factors such as lighting, the original model did not accurately detect the nose undercarriage with higher exposure. In contrast, the improved model saw all undercarriages with better precision location. Figure 11 shows the detection of an aeroplane during take-off, where the optimized model detects targets not detected by the original model with higher confidence. Figure 12 shows the detection inside the airfield, that YOLOv5-RSC did not misdetect the tires of the tractor, and that the detection precision has been improved, proving that the model proposed in this paper has stronger robustness and generalization ability.

**Figure 13:**
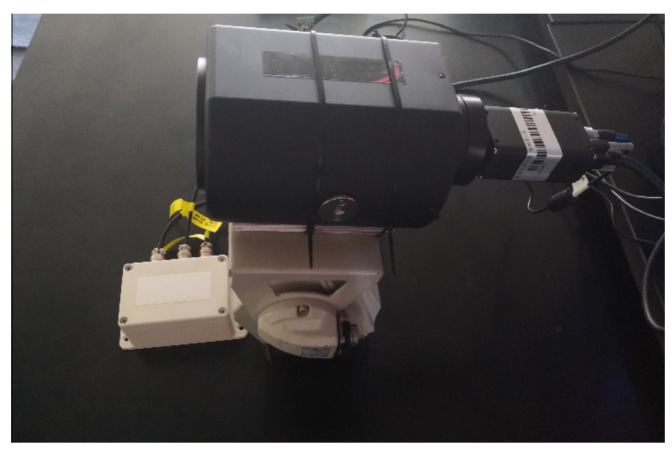
Undercarriage detection system.

**Figure 14:**
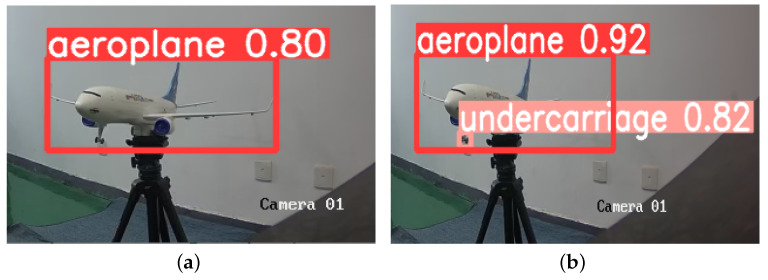
Close range detection. (**a**) YOLOv5 test results; (**b**) YOLOv5-RSC test results.

**Figure 15:**
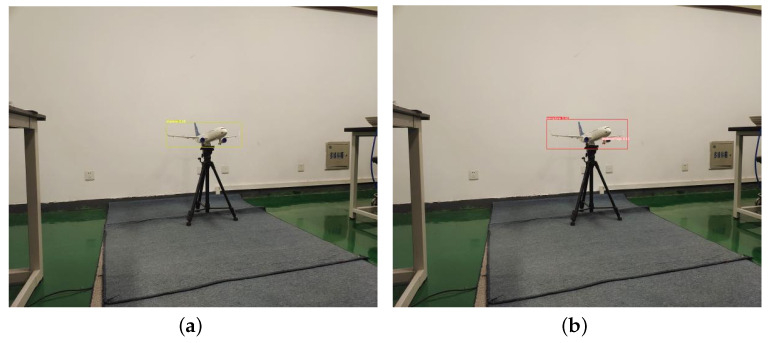
Medium and long distance detection. (**a**) YOLOv5 test results; (**b**) YOLOv5-RSC test results.

In order to verify the actual detection effect of the YOLOv5-RSC model, this paper uses a Basler camera, SPACECOM lens, and two-axis gimbal to build an undercarriage detection system based on the GPU mentioned above, CPU, and other hardware, as shown in Figure 13. Utilizing this system to implement the enhanced model in real-world scenarios, we present the experimental results in Figure 14 and Figure 15. From the experimental results, it can be seen that the detection precision of the improved model is higher than that of the original model, and it can also detect that the original model can not detect the targets. The superiority of the YOLOv5-RSC model over the original model is evident in both evaluation indices and practical applications. It has stronger generalization ability, robustness in detecting small and dense targets, and stronger detection performance.

## 5. Conclusions

This study presents an aeroplane and undercarriage detection model, YOLOv5-RSC, which builds upon the improved YOLOv5 algorithm. We seamlessly integrate this model with the Basler Camera Sensor Experimental Platform, aiming to tackle the challenges inherent in detecting small target aeroplanes. Firstly, we incorporate RFBNet into the feature extraction network to enhance the network’s ability to extract deep features and generate more robust features. Secondly, we introduce a coordinate attention mechanism to the feature fusion network, allowing it to extract key information from the image while disregarding irrelevant details. Finally, the SIOU bounding box loss function is introduced to expedite model convergence and enhance detection efficiency. To assess the model’s effectiveness, we curate aeroplane and undercarriage datasets using the data enhancement and annotation tool LabelImg. Subsequently, we conduct ablation experiments and comparison studies with other classical deep learning detection algorithms. The experimental results reveal that the YOLOv5-RSC model proposed in this paper achieves a precision and recall rate of 89.5% and 82.1%, respectively, an mAP of 86.4%, and an FPS of 89.2. These metrics significantly surpass those of the SSD and Faster R-CNN algorithms in terms of performance. Compared with the original YOLOv5 algorithm, the FPS has decreased by 10.8, but the other indicators show improvement. The results affirm that the model demonstrates robust detection capabilities and performs real-time detection tasks, signifying practical significance. Additionally, it offers insights into integrating sensor technology with image detection tasks to create an “intelligent image sensor”.

## Figures and Tables

**Table 1:** Experimental results of ablation of coordinate attention.

Experimental Group	Precision	Recall	mAP@0.5
	86.9%	80.0%	82.7%
a	86.7%	79.5%	82.7%
b	87.7%	80.5%	84.8%
c	86.5%	80.3%	84.4%

**Table 2:** Confusion matrix.

	True	False
Positive	TP	FP
Negative	TN	FN

**Table 3:** Performance comparison of different models.

Model	Aeroplane /AP	Undercarriage /AP	mAP@0.5	FPS/ (Frame/s)
Faster R-CNN	60.8%	58.6%	59.7%	7.3
SSD	64.4%	59.8%	62.1%	20.6
RetinaNet	68.1%	60.4%	64.3%	25.4
YOLOv4	76.7%	62.3%	69.5%	76.3
YOLOv5s	90.4%	75.1%	82.7%	100.0
YOLOv5-RSC	92.4%	80.5%	86.4%	89.2

**Table 4:** Comparison results with other undercarriage detection model.

Model	Aeroplane /AP	Undercarriage /AP	mAP@0.5	FPS/ (Frame/s)
Gao’s model	76.0%	68.0%	71.9%	20.0
Our model	92.4%	80.5%	86.4%	89.2

**Table 5:** Results of ablation experiments.

Model	Precision	Recall	mAP@0.5	FPS/(Frame/s)
YOLOv5s	86.9%	80.0%	82.7%	100.0
YOLOv5 + BasicRFB	87.7%	80.9%	85.0%	93.1
YOLOv5 + SIOU	87.5%	81.1%	84.7%	100.0
YOLOv5 + CA	87.7%	80.5%	84.8%	94.3
YOLOv5 + BasicRFB + CA	89.2%	80.5%	86.1%	90.0
YOLOv5 + BasicRFB + SIOU	88.6%	81.5%	86.0%	92.8
YOLOv5-RSC	89.5%	82.1%	86.4%	89.2

## Data Availability

Data are contained within the article.
